# Proton Pump Inhibitors Use and the Risk of Pancreatic Cancer: Evidence from Eleven Epidemiological Studies, Comprising 1.5 Million Individuals

**DOI:** 10.3390/cancers14215357

**Published:** 2022-10-30

**Authors:** Tahmina Nasrin Poly, Md. Mohaimenul Islam, Bruno Andreas Walther, Ming-Chin Lin, Yu-Chuan (Jack) Li

**Affiliations:** 1 Graduate Institute of Biomedical Informatics, College of Medical Science and Technology, Taipei Medical University, Taipei 110301, Taiwan; 2 International Center for Health Information Technology (ICHIT), Taipei Medical University, Taipei 110301, Taiwan; 3 Research Center of Big Data and Meta-Analysis, Wan Fang Hospital, Taipei Medical University, Taipei 110301, Taiwan; 4 Alfred-Wegener-Institut Helmholtz-Zentrum für Polar-und Meeresforschung, Am Handelshafen 12, D-27570 Bremerhaven, Germany; 5 Department of Neurosurgery, Shuang Ho Hospital, Taipei Medical University, New Taipei City 235041, Taiwan; 6 Department of Dermatology, Wan Fang Hospital, Taipei 116, Taiwan

**Keywords:** proton pump inhibitor, pancreatic cancer, omeprazole, meta-analysis

## Abstract

**Simple Summary:**

Proton pump inhibitors are commonly prescribed medications for gastrointestinal disorders, which bring gastric acid down to normal levels. However, the effects of PPI on pancreatic risk remain unclear. Therefore, we conducted a systematic review and meta-analysis to investigate the association between PPI use and pancreatic cancer. The overall combined estimate suggested that PPI therapy was significantly associated with an increased risk of pancreatic cancer (RR_adj._ 1.63, 95%CI: 1.19–2.22, *p* = 0.002). However, this effect might be biased due to users’ definitions, exposure periods, and other confounding factors. Large epidemiological studies with controlled bias are therefore warranted to confirm or refute the association found in this study. Considering the possible carcinogenic effect of PPI, physicians should be vigilant when prescribing high-dose or long-term PPI.

**Abstract:**

Previous epidemiological studies have shown that proton pump inhibitor (PPI) may modify the risk of pancreatic cancer. We conducted an updated systematic review and meta-analysis of observational studies assessing the effect of PPI on pancreatic cancer. PubMed, Embase, Scopus, and Web of Science were searched for studies published between 1 January 2000, and 1 May 2022. We only included studies that assessed exposure to PPI, reported pancreatic cancer outcomes, and provided effect sizes (hazard ratio or odds ratio) with 95% confidence intervals (CIs). We calculated an adjusted pooled risk ratio (RR) with 95%CIs using the random-effects model. Eleven studies (eight case–control and three cohorts) that reported 51,629 cases of pancreatic cancer were included. PPI was significantly associated with a 63% increased risk of pancreatic cancer (RR_adj._ 1.63, 95%CI: 1.19–2.22, *p* = 0.002). Subgroup analysis showed that the pooled RR for rabeprazole and lansoprazole was 4.08 (95%CI: 0.61–26.92) and 2.25 (95%CI: 0.83–6.07), respectively. Moreover, the risk of pancreatic cancer was established for both the Asian (RR_adj._ 1.37, 95%CI: 0.98–1.81) and Western populations (RR_adj_.2.76, 95%CI: 0.79–9.56). The findings of this updated meta-analysis demonstrate that the use of PPI was associated with an increased risk of pancreatic cancer. Future studies are needed to improve the quality of evidence through better verification of PPI status (e.g., patient selection, duration, and dosages), adjusting for possible confounders, and ensuring long-term follow-up.

## 1. Introduction

With an annual incidence of approximately 0.5 million, pancreatic cancer is the 12th most common cancer and the seventh most frequent cause of cancer death with >0.43 million deaths annually [1]. The age-standardized incidence and mortality rate of pancreatic cancer is highest in Europe, followed by North America and Oceania [2,3]. The 5-year survival rate of pancreatic cancer varies in different counties; unfortunately, it is still less than 12% [4,5]. Early detection of pancreatic cancer and proper treatments may improve the outcomes. Numerous studies have extensively investigated the etiology of pancreatic cancer and identified several modifiable (e.g., smoking, alcohol consumption, and obesity) and non-modifiable risk factors (e.g., age, race and ethnicity, family history and genetics) [6,7,8]. The current strategy to minimize the risk of pancreatic cancer includes changing behavior, screening high-risk patients, and identifying cancer-inducing agents.

Proton-pump inhibitors (PPIs) are first-line medications in the clinical practice for the management of acid-related disorders. Since PPIs are often considered safe over-the-counter medications; therefore, less attention is paid by healthcare providers. Nowadays, adverse effects of long-term use of PPIs are gaining increasing attention, especially in the risk of gastric [9], colorectal [10], and oesophageal cancer [11]. Epidemiological studies also highlighted the association between PPIs and pancreatic cancer risk, but there was a discrepancy among the finding. Peng et al. [12] conducted a study regarding the relationship between pancreatic cancer and PPI users and indicated an increased risk of pancreatic cancer among PPIs users. However, Lassalle et al. found no increased risk of dementia among PPIs users at all [13]. Hence, the association between PPIs and pancreatic cancer remains unclear before re-evaluating the pooling effects.

Therefore, we conducted an updated systematic review and meta-analysis of existing observational studies that evaluated the association between PPI and the risk of developing pancreatic cancer.

## 2. Methods

We followed the guidance provided by the Cochrane Handbook [14]; thus, our study reports according to the Meta-analysis of Observational Studies in Epidemiology guidelines [15].

**Data Sources and Search Strategy:** PubMed, Embase, Scopus, and Web of Science were searched for published studies related to PPIs and pancreatic cancer between 1 January 2000, and 1 May 2022. Search terms used included “proton-pump inhibitor(s)” OR “omeprazole” OR “pantoprazole” OR “lansoprazole” OR “esomeprazole” OR “rabeprazole” AND “pancreatic cancer” OR “neoplasm(s)” OR “pancreatic malignancy(ies)”. The titles and abstracts of retrieved studies were screened to exclude irrelevant studies. The full texts of the remaining studies were examined to extract information that evaluated the effects of PPI on pancreatic cancer risk. The reference lists of the retrieved studies were also examined to obtain additional studies.

**Inclusion and Exclusion Criteria:** We included all observational (cohort or case–control) studies that assessed exposure to PPI and risk of pancreatic cancer. All the observational studies needed to be published in English and provide effect sizes (OR/HR) with 95%CIs. Included studies were also required to provide clear information regarding the patients’ characteristics, inclusion, and exclusion criteria. Studies were excluded if they were reviews, editorials, case-reports, or letters to editors without data description.

**Data Extraction:** Two authors (TNP and MMI) developed screening guidelines and examined the appropriateness of all included studies for inclusion. The two authors extracted the following information from each study: (i) author’s first and last name, publication year, country of the participants; (ii) study design; (iii) number of participants, age, gender; (iv) inclusion and exclusion criteria, adjusted confounding factors; (v) definition of PPI exposure, long-term PPI use, individual PPI exposure; (vi) effect sizes (OR/HR), and 95%CIs.

**Quality Assessment:** The same two authors independently examined the quality of all included studies using the Newcastle-Ottawa Scale (NOS) recommended by the Cochrane library [16,17]. The NOS uses three parameters to assess the quality of each study: selection, comparability, and outcome (cohort studies) or exposure (case–control studies). The NOS has a maximum of nine points which are given according to the following criteria: (1) selection: a maximum of four points, (2) comparability: two points, and (3) exposure/outcome: three points. A study which receives nine points is categorized as “high” quality, seven to eight points as “medium” quality, and less than seven as “low” quality. Any discrepancy in this evaluation between the two authors was resolved by reexamination of the original study and discussion with a third author.

**Data Analysis:** The random-effects model (DerSimonian and Laird) was used to calculate the pooled RR and 95% CI. The heterogeneity between studies was estimated using two different methods. First, we calculated Cochran’s Q statistic for assessing heterogeneity. It tests the null hypothesis that all included studies obtain the same underlying magnitude of effect. The Q statistical test usually has insufficient power to distinguish a moderate degree of heterogeneity [18]. A *p*-value of <0.05 was considered to indicate significant heterogeneity. Second, we also calculated the I^2^ statistic to determine the proportion of total variation between studies due to heterogeneity rather than chance. In this case, values of I^2^ of <25%, 25 ~ <50%, 50 ~ <75%, and >75% were categorized as null, low, moderate, and high heterogeneity, respectively [19,20,21]. We investigated the presence of publication bias using Egger’s regression test (publication bias is considered if *p* ≤ 0.05). We also used the funnel plot of the logarithm of RRs versus their standard errors. All analyses were performed using the Comprehensive Meta-analysis Software (CMA) version V3 (Biostat Inc, Englewood, NJ, USA). All statistical tests were two-sided, and a *p*-value < 0.05 was considered to be statistically significant.

## 3. Results

**Search Results:** Our primary search selected a total of 482 studies. After reviewing the titles and abstracts of these 482 studies, 464 were excluded as ineligible as they were duplicates, reviews, case-reports, letters, and others which did not meet the prespecified inclusion criteria. The remaining 18 studies went through full-text evaluation. Of these, a further seven studies were excluded for reasons presented in Supplementary Figure S1. Consequently, the remaining 11 studies were included in our meta-analysis [12,13,22,23,24,25,26,27,28,29,30].

**Study Characteristics:** The characteristics of these 11 studies are presented in Supplementary Table S1. The 11 studies represented eight case–control and three cohort studies involving a total of 1,556,182 participants and 51,629 cases of pancreatic cancer. The 11 studies were published between 2012 and 2021. Eight studies were conducted in Western countries [13,22,23,24,26,27,29,30] (four in Europe, three in North America, and one was a collaboration of multi-centers across Europe, North America, and Australia), and three studies were conducted in Asian populations [12,25,28] (two in Taiwan, one in South Korea).

**Quality of Included Studies:** Two cohort studies had a NOS score of 9, and the remaining cohort study had a score of 8. For the case–control studies, four out of eight studies (50%) were of low quality (NOS score < 7), with an average NOS score of 7.27.

**Figure 1:**
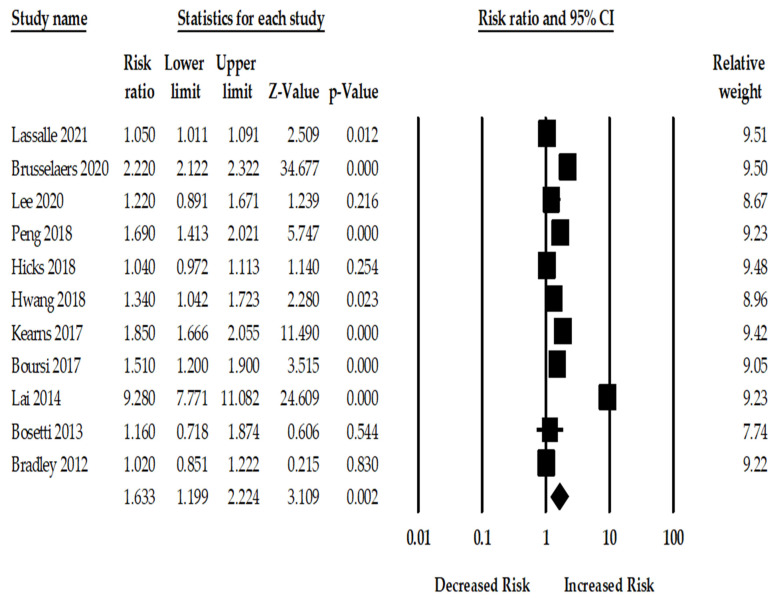
Forest plot of association between PPI and pancreatic cancer risk for eleven studies [12,13,22,23,24,25,26,27,28,29,30].

**PPI Use and the Risk of Pancreatic Cancer:** Among the included 11 studies, the use of PPI was associated with a statistically significant 63% increase in pancreatic cancer (RR_adj._ 1.63; 95%CI: 1.19–2.22, *p* = 0.002). There was, however, considerable heterogeneity across the studies (Q = 1172.45, I^2^ = 99.14, τ^2^ = 0.26, *p* < 0.001). Figure 1 shows the risk of pancreatic cancer among PPI users for the 11 included studies.

**Subgroup Analysis:** We conducted subgroup analyses of the included 11 studies based on study design, location, adjusted factors, number of participants, study quality, and individual PPIs use (Table 1). The adjusted pooled analysis of the eight case–control studies also resulted in a significant association between PPI use and the risk of pancreatic cancer (RR_adj._ 1.62, 95%CI: 1.12–2.34, *p* = 0.01, Q = 656.94, I^2^ = 98.73%). The overall pooled analysis of the three cohort studies demonstrated a significant positive association with pancreatic cancer (RR_adj._ 1.67, 95%CI: 1.17–2.39, *p* = 0.004, Q = 24.57, I^2^ = 91.86%). However, the overall pooled analysis of individual PPI use showed a non-significant association with pancreatic cancer.

**Sensitivity Analysis:** Since the overall findings had high heterogeneity (I^2^ = 99.14%, *p* < 0.001), we performed a sensitivity analysis. In order to assess the overall impact of a single study on pancreatic cancer risk, a sensitivity analysis was conducted by excluding studies one by one. First, we excluded Bosetti et al.’s [29] study from the primary analysis because the main objective of the study was not directly related to PPI use and pancreatic cancer risk. The overall pooled risk of pancreatic cancer was 1.68 (95%CI:1.21–2.32, *p* = 0.002, I^2^ = 99.23%). Second, Boursi et al.’s [27] study was excluded because they assessed the risk of pancreatic cancer for those with new-onset diabetes. The adjusted pooled RR of developing pancreatic cancer among PPI users was 1.70 (95%CI:1.20–2.39, *p* = 0.002, I^2^ = 99.31%). However, the sensitivity analysis did not substantially change the pooled effect and the level of heterogeneity.

**Figure 2:**
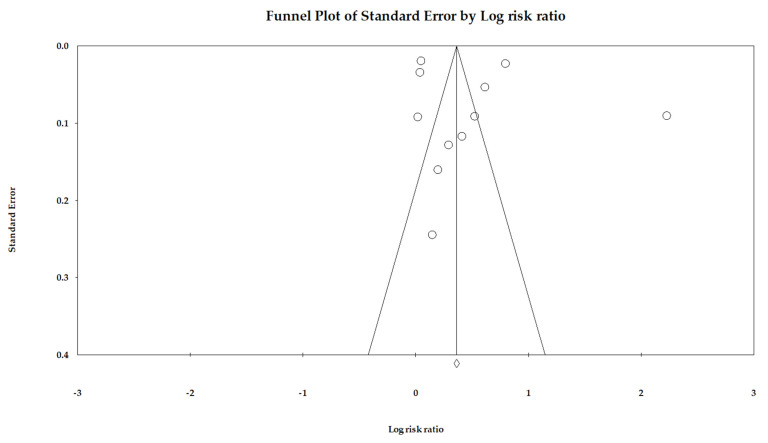
Funnel plot.

**Publication Bias:** We used Egger’s regression to detect overall publication bias and also generated Begg’s funnel plots (Figure 2). However, the distribution of included studies was relatively symmetric, indicating very little publication bias (*p* = 0.55). We utilized Duval and Tweedie’s trim-and-fill methods, and the adjusted RR was 1.43 (95% CI: 1.40–1.47); hence, the impact of this bias was probably close to null. 

## 4. Discussion

This is an updated meta-analysis of eleven observational studies involving more than 1.5 million individuals, which evaluated the effect of PPI use on the risk of pancreatic cancer. Our meta-analysis found a moderately increased risk of pancreatic cancer in people using PPI. However, this association was not statistically significant when stratified by region.

Previous meta-analyses of observational studies on PPI uses and the risk of pancreatic cancer reported that there was a positive link between them [31,32,33], which is consistent with our findings. Laoveeravat et al. [32] included seven studies with a total of 546,199 participants. Compared to patients who did not take PPI, the pooled RR of pancreatic cancer in patients receiving PPI was 1.73 (95%CI: 1.16–2.57). However, they did not conduct any subgroup and sensitivity analyses. Alkhushaym et al. [31] evaluated the effect of PPI on pancreatic cancer with a total of 700,178 participants. The findings of their study showed that PPI use was associated with a 75% increased risk of pancreatic cancer (pooled RR, 1.75 95%CI: 1.12–2.72), with high statistical significance (*p* < 0.001) but also high heterogeneity (I^2^ = 99%). This study also lacked subgroup analyses. Furthermore, Hong et al. [33] conducted a meta-analysis to determine the risk of pancreatic cancer among PPI users. They included ten observational studies with 948,782 participants. A positive association between PPI use and pancreatic cancer was observed (pooled RR 1.69, 95%CI: 1.20–2.40, I^2^ = 98.75%). Their study also did not provide subgroup analyses or any information regarding doses and duration. In contrast, our updated meta-analysis used a higher number of studies and conducted comprehensive subgroup analyses to assess the differential effects of PPI use on pancreatic cancer. Furthermore, our study showed the effect sizes with several confounding factors that were not addressed by previous studies.

The etiology of pancreatic cancer is multifactorial; age, sex, geographical location, genetic and behavioral factors are key contributors [34,35]. Although the exact biological pathway remains unidentified, there are several plausible biological pathways which could explain the link between PPI use and pancreatic cancer. First, gastrin has a dual role which is related to meal-induced gastric acid secretion and as a trophic hormone for epithelial and enterochromaffin cells. However, gastrin and their receptor (CCK-B/gastrin-like receptor) have a shared common link to develop pancreatic cancer [36]. Second, long-term use of PPI induces hypergasterinemia which could also be a potential factor in the development of pancreatic cancer [37,38]. Third, the reduction in gastric acid due to PPI use instigates bacterial growth and secretion of nitorsamides, which can be responsible for increasing pancreatic cell overgrowth [39,40]. Finally, PPI use can impair vitamin B_12_ absorption [41] because B_12_ plays a key role in pancreatic cancer as reported in previous studies [42,43].

In the subgroup analysis discerning between study populations, the risk of pancreatic cancer was higher in the Asian than in the Western population. Regional differences are always complicated. Previous evidence demonstrated that many physiological determinants, such as genetic factors [44,45], lifestyle (e.g., eating habits, smoking, alcohol, physical activity) [46] are related to these variations. Additionally, environmental factors (pollution, socioeconomic status, and stress) [8,47] and public health services may also contribute to these differences [4,48]. However, gradual improvement of gastric disorder symptoms, early screening, and diagnosis of pancreatic cancer risk factors can improve the situation. The risk of pancreatic cancer among races cannot be fully explained by the known and suspected risk factors [49]. Our study findings for geographical differences have potential limitations of the small number of available studies. The statistically insignificant association existed both in Asia and the Western population. Thus, caution is needed when interpreting these findings.

Subgroup analyses showed an insignificant association among the studies adjusted with smoking status. Previous evidence highlighted smoking as a recognized risk factor for pancreatic cancer [50,51]. An insignificant association can be explained by a limited number of studies (only three studies were used to pool effect size). The pooled effect size of low-quality studies showed a high risk of pancreatic cancer among PPI users than moderate and high-quality studies. It is because the patient selection and potential risk of bias are often tried to control in high and moderate-quality of studies than low-quality studies. Finally, our study shows that all kinds of PPI are associated with an increased risk of pancreatic cancer, although the relationship between them was statistically insignificant. More studies with control patient selection are needed to measure possible associations.

## 5. Implications for Practice and Research

Due to limited evidence and lack of high-quality studies on the effect of PPI use on pancreatic cancer, we believe that the following aspects are needed to be investigated in future studies and in the real-world clinical practice.

(i) Study design: All the studies can use the Strengthening the Reporting of Observational Studies in Epidemiology (STROBE) guidelines [52] to follow a standard study design and generate quality evidence. It is well known that randomized control trials cannot always answer all the questions, and they take a long time to conduct. However, observational studies have considerable importance in assessing the benefits and harms of medical interventions. Indeed, observational studies have great potential to identify the unwanted consequences of any drug treatment and are more likely to provide an appropriate direction of what is happening in real-world clinical practice [53]. The credibility of the evidence of the observational studies depends on the strengths and weaknesses in the study design, conduct, and analysis. Every study needs to properly define patient selection such as what type of PPI users they were, how long patients were taking PPI, and what were the dose limits, and what was the follow-up duration. Future studies should also classify patients into several groups, such as continuous, intermittent, and low users. Moreover, all PPI cannot have the same effects; therefore, all the studies should provide individual PPI effect on pancreatic cancer. It would be helpful for physicians and patients to consider PPI for treating or disease management. For example, if omeprazole is safer than other PPI, physician could consider omeprazole for the patients with GERD.

(ii) PPI dose and duration: All studies may provide information regarding the short and long-term users and the risk of pancreatic cancer. For example, physicians often prescribe PPIs for 15 days to treat common symptoms of GERD. However, physicians also consider the long-term use of PPIs for patients with severe erosive esophagitis or Barrett’s esophagus. However, there is no appropriate formula on how to define long-term PPI use [54], and the definitions of long-term use of PPIs always vary between studies. Significant differences in definitions of short and long-term use of PPIs make the situation complex to the pooled natural effect of extended continuous and discontinuous long-term use of PPI [55]. However, a uniform definition is essential in the clinical context. Future studies could calculate the effect of PPI use every six months (e.g., six months, 1-year, 1.5 years, and so forth). Moreover, included studies provided information regarding daily dose and the risk of pancreatic cancer but classification varied from study to study. For example, one study evaluated <30 DDD, 30–180 DDD, and >180 DDD [13]; however, another study assessed the risk of pancreatic cancer among PPI users with <30 DDD, 30–65 DDD, and 65–150 DDD [12]. Furthermore, they did not provide any information on how they calculated DDD. Therefore, it is hard to reach a clinical conclusion about dose and duration when a meta-analysis is conducted using various studies’ information. Therefore, a standard protocol is warranted to summarize the effect of various doses and durations.

(iii) Confounding bias: In the real-world clinical setting, randomized control trials are often considered a “gold standard” method because they control for potential risk bias. However, confounding factors/effects occur in observational studies and change the outcome of interests, either directly or indirectly. These biases can weaken or strengthen or alter the actual association. All included studies adjusted confounding factors except for two studies [27,29]. However, confounding factors differed from study to study. In the future, studies can address all possible confounding factors such as age >60 years, chronic pancreatitis, diabetes, and obesity. Possible confounding factors can also be identified from previous studies. Future studies can measure similar adjustment, matching, or stratification variables in the same way. So, when a meta-analysis will be conducted, it could evaluate the effect of all possible confounding factors and assess the actual effect size of pancreatic cancer with PPI.

**Limitations:** Our meta-analysis has several limitations. First, all included studies in this meta-analysis were observational studies. Observational studies are often susceptible to uncontrolled biases, even if they are well designed, which may weaken the actual quality of the analysis. Second, two-thirds of the included studies in our meta-analysis came from Western countries, and only three studies were from Asia, even though the prevalence and mortality of pancreatic cancer have been rapidly increasing in Asia [34,49]. Third, we were unable to present the association between PPI and pancreatic cancer based on various duration [12,23] and dosages [12,13,23,24]. Finally, the heterogeneity among the studies was high, and several studies did not provide detailed information regarding confounding factors. Nevertheless, we used the random effect models and showed the effect sizes for different confounding factors.

## 6. Conclusions

We conducted an updated meta-analysis of the association between PPI use and pancreatic cancer risk in more than 1.5 million participants. The findings of this study show that PPI use was significantly associated with an increased risk of pancreatic cancer. Our robust analyses contribute to a better understanding of PPI use and pancreatic cancer risk. Since PPI is associated with an increased risk of pancreatic cancer, physicians should be cautious when prescribing PPI for GERD patients. More epidemiological and mechanistic studies are warranted to further examine the relationship between PPIs and the risk of pancreatic cancer. Future studies should also focus on adjusting possible confounders and identifying the probable mechanisms underlying this association.

## Figures and Tables

**Table 1:** Subgroup analysis of all studies.

Subgroup	No. of Study	Effect Size	95% CI	*p*-Value	*I* ^2^	Q-Value	*p*-Value	τ^2^
All	11	1.63	1.19–2.22	0.002	99.14	1172.45	<0.001	0.26
**Study design**								
Case-control	8	1.62	1.12–2.34	0.01	98.73	656.94	<0.001	0.27
Cohort	3	1.67	1.17–2.39	0.004	91.86	24.57	<0.001	0.08
**Region**								
Western	8	1.37	0.98–1.81	0.06	99.04	734.89	<0.001	0.17
Asian	3	2.76	0.79–9.56	0.10	99.14	233.21	<0.001	1.19
**Methodological quality**								
High	3	1.26	0.97–1.62	0.07	84.13	12.60	0.002	0.04
Moderate	4	1.30	0.78–2.17	0.30	99.19	373.68	<0.001	0.26
Low	4	2.43	1.03–5.73	0.04	98.88	269.71	<0.001	0.74
**Sample size**								
≤10,000	5	1.87	0.73–4.80	0.18	98.85	350.02	<0.001	1.12
>10,000	6	1.44	1.02–2.04	0.03	99.30	722.77	<0.001	0.18
Adjusted for age								
Yes	11	1.63	1.19–2.22	0.002	99.14	1172.45	<0.001	0.26
Adjusted for smoking status								
Yes	3	1.45	0.83–2.53	0.18	97.48	79.42	<0.001	0.23
No	8	1.70	1.17–2.48	0.005	98.93	658.24	<0.001	0.27
Adjusted for chronic pancreatitis								
Yes	7	1.75	1.16–2.63	0.007	99.47	1147.15	<0.001	0.29
No	4	1.49	1.18–1.87	0.001	70.63	10.21	0.01	0.03
Adjusted for diabetes								
Yes	8	1.70	1.17–2.46	0.005	99.40	1168.32	<0.001	0.27
No	3	1.56	1.34–1.82	<0.001	12.47	2.28	0.31	0.002
Adjusted for *H. pylori*								
Yes	2	1.04	1.01–1.08	0.006	0	0.05	0.80	0
No	9	1.81	1.28–2.55	0.001	97.91	384.49	<0.001	0.26
Adjusted for obesity								
Yes	3	2.61	0.97–6.97	0.05	99.67	622.32	<0.001	0.75
No	8	1.36	0.9–1.89	0.06	98.16	380.61	<0.001	0.20

## Data Availability

The data presented in this study are available in this article (and Supplementary Material).

## Supplementary Materials

The following supporting information can be downloaded at: https://www.mdpi.com/article/10.3390/cancers14215357/s1, Figure S1: Flow diagram of the study search and selection for evaluating the risk of pancreatic cancer among PPI users; Table S1: Characteristics of the 11 studies assessing the risk of pancreatic cancer with PPI use.
