# Supplementary material for: Proton Pump Inhibitors Use and the Risk of Pancreatic Cancer: Evidence from Eleven Epidemiological Studies, Comprising 1.5 Million Individuals
Source: Cancers (Basel). 2022 Oct 30;14(21):5357. doi: 10.3390/cancers14215357 (PMC9658965; doi:10.3390/cancers14215357)
Supplement: Supplementary file 1 [file cancers-14-05357-s001.zip › cancers-1874067-supplementary.pdf]

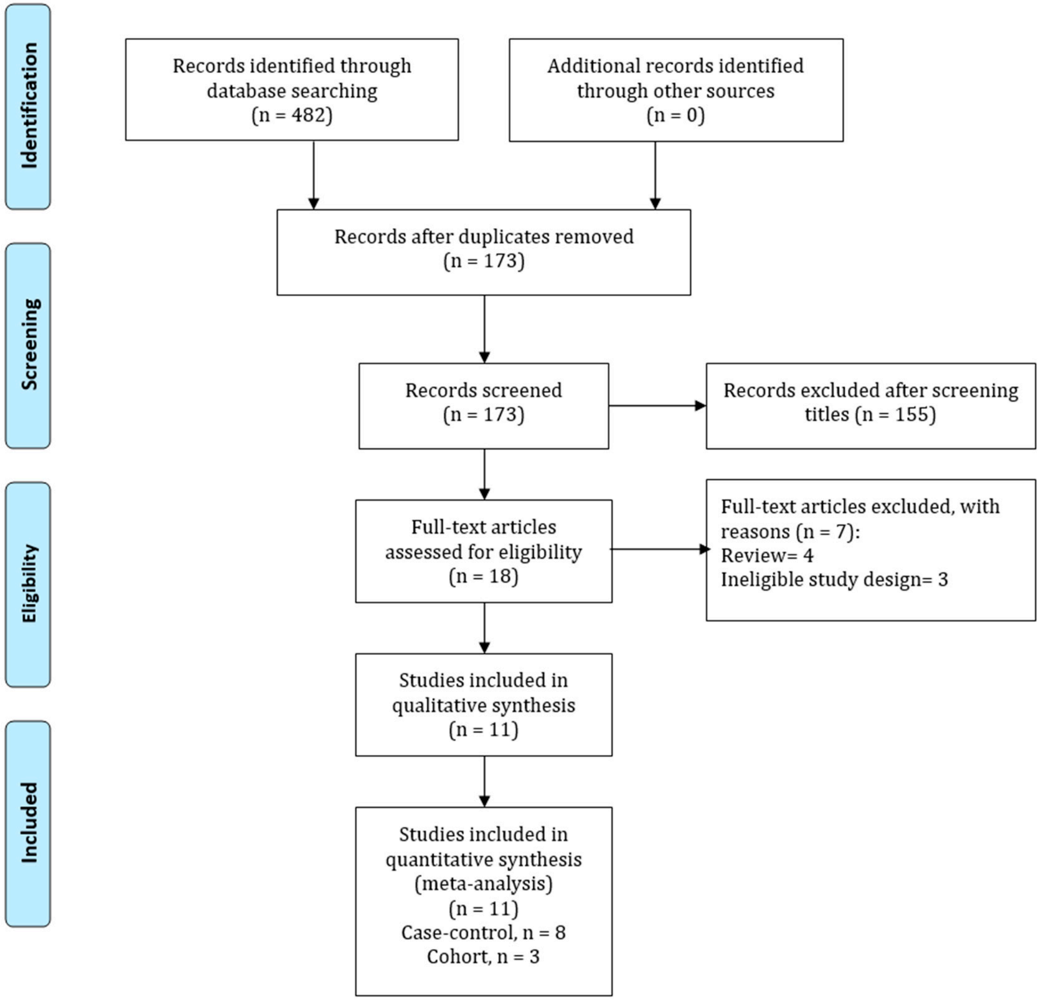

Supplementary Figure S1. Flow diagram of the study search and selection for evaluating the risk of pancreatic cancer among PPI users.

Supplementary Table S1. Characteristics of the 11 studies assessing the risk of pancreatic cancer with PPI use.

Author	Country	Design	Duration	TP	Pancreatic cancer	Age	Male	Inclusion criteria for pancreatic cancer	Adjusted factors	NOS
Lassalle-2021	France	C-C	2014-2018	23321/75937	23321	69.8/70.0	51.7/51.8	ICD	Age, History of diabetes, obesity, pancreatitis, hepatitis B or C, *H. pylori* eradication, statin	9
Brusselaers-2020	Sweden	Cohort	2005-2012	796492	1733	67.8/67.3	41.5	ICD	Age, gender, gastric acid suppressant, diabetes	8
Lee -2020	USA	C-C	1996-2016	567/4870	567	N/A	50.6/51.5	ICD	Alcohol, age, smoking, gastric cancer, BMI, chronic pancreatitis, *H. pylori*, dysplasia, diabetes, liver disease, colitis, etc.	8
Peng-2018	Taiwan	C-C	2006-2011	1087/1087	1087	68.3/67.4	60.9/59.8	ICD	Age, chronic pancreatitis, CAD, COPD	6
Hicks-2018	Denmark	C-C	2000-2015	6921/34605	6921	N/A	N/A	ICD	Age, gender, diabetes, COPD, gallstones, chronic pancreatitis, peptic ulcer, hepatitis B and	7

									C, aspirin, statin, etc.	
Hwang- 2018	Korea	Cohort	2002-2013	453655	3086	NA	53.5	ICD	Age, gender, myocardial infarction, CHF, dementia, pulmonary disease, peptic ulcer, liver disease, etc.	9
D.Kearns-2017	UK	C-C	1995-2013	4113/16072	4113	70.9/71.1	51.4/51.1	ICD	Age, sex, Diabetes, smoking, alcohol, obesity	6
Boursi-2017	UK	Cohort	1995-2013	109385	390	62.7	53.6	ICD	Age, sex	9
Lai - 2014	Taiwan	C-C	2000-2010	977/3908	977	68.38/68.11	60.59/60.59	ICD	Age, sex, Chronic pancreatitis, diabetes, obesity, H2RA, statin, non-statin, both ASA and COX2 inhibitor	6
Bosetti-2013	USA + Canada + Australia	C-C	N/A	4717/9374	4717	NA	56.5/56.6	ICD	Age, sex, comorbidities	5
Bradley- 2012	UK	C-C	1995-2006	4717/7954	4717	57.3	53.37	ICD	Age, sex, smoking, BMI, alcohol, chronic pancreatitis, NSAIDs, HRT, diabetes, prior cancer	7

Abbreviations: *UK: United Kingdom, USA: United States of America, C-C: Case-control, TP: Total population, ICD: International Classification of Diseases, N/A: Not available, NOS: Newcastle-Ottawa Scale, BMI: Body Mass Index, CAD: Coronary Artery Disease, COPD: Chronic Obstructive Pulmonary Disease, CHF: Coronary Heart Disease, H2RA: H2 Receptor Antagonist, NSAIDs: Non-steroidal Anti-inflammatory Drugs, HRT: Hormone Replacement Therapy*
